# Rhubarb Enema Attenuates Renal Tubulointerstitial Fibrosis in 5/6 Nephrectomized Rats by Alleviating Indoxyl Sulfate Overload

**DOI:** 10.1371/journal.pone.0144726

**Published:** 2015-12-15

**Authors:** Zhaoyu Lu, Yuqun Zeng, Fuhua Lu, Xusheng Liu, Chuan Zou

**Affiliations:** National Key Unit of Clinical Research of TCM on Chronic Kidney Diseases, Key Unit of Kidney Diseases, Guangdong Provincial Hospital of TCM, the Second Affiliated Hospital of Guangzhou University of Chinese Medicine, Guangzhou, 510120, P.R.China; Emory University, UNITED STATES

## Abstract

**Aim:**

To investigate the effects of rhubarb enema treatment using a 5/6 nephrectomized rat model and study its mechanisms.

**Methods:**

Twenty-eight Sprague Dawley rats were divided into three groups: sham operation group (n = 8), 5/6 nephrectomized (5/6Nx) (n = 10), and 5/6Nx with rhubarb enema treatment (n = 10). The rhubarb enema was continuous for 1.0 month. Serum creatinine, serum indoxyl sulfate (IS) level, renal pathology, tubulointerstitial fibrosis, and renal oxidative stress were assessed.

**Results:**

5/6Nx rats showed increasing levels of serum creatinine and severe pathological lesions. Their serum creatinine levels obviously decreased after rhubarb enema treatment (*P* < 0.05 vs 5/6Nx group). The administration of rhubarb enema attenuated the histopathological changes in 5/6Nx rats. In addition, 5/6Nx rats showed an enhanced extent of tubulointerstitial fibrosis compared with sham rats, and administration of rhubarb enema to 5/6Nx rats ameliorated tubulointerstitial fibrosis. 5/6Nx rats showed increased serum levels of IS, renal oxidative stress, and NF-κB compared with sham rats, whereas administration of rhubarb enema to 5/6Nx rats decreased serum levels of IS, renal oxidative stress, and NF-κB levels.

**Conclusion:**

Rhubarb enema treatment ameliorates tubulointerstitial fibrosis in the kidneys of 5/6Nx rats, most likely by alleviating IS overload and reducing kidney oxidative stress and inflammatory injury.

## Introduction

Chronic kidney disease (CKD) has become a worldwide health and social problem. The prevalence of CKD has increased to 14.4% in the United States [1] and is 10.8% in China [2]; consequently, retarding the progression of CKD into end-stage renal disease (ESRD) would be beneficial to patients, the economy and the healthcare system.

Clinical evidence has proved that angiotensin-converting enzyme inhibitor and angiotensin receptor blocker act on lowering blood pressure, decreasing proteinuria, and retarding CKD progression; however, the effectiveness is not found in every CKD patient, especially in non-dialysis patients with stage 5 CKD, or in cases of hyperkalemia or acute kidney injury. Other possible approaches to protect the kidneys, such as restricting protein intake, lowering lipids, intensively controlling blood glucose, and quitting smoking, require more evidence of their effectiveness [3]. There is still a lack of effective methods by which to reduce the progression of chronic kidney diseases, but new approaches to retard CKD progression have been globally explored throughout kidney disease academia.

It is well known that the accumulation of uremic toxins, especially IS, is a key mechanism in CKD progression [4, 5]. Recent studies discovered that the colon is an important organ from which uremic toxins are generated. Enterogenous uremic toxins such as indoxyl sulfate (IS) in the blood not only promote the progression of CKD, but are also closely related to the mortality of CKD patients [6].

An enema with a rhubarb-based compound decoction or a Chinese patent drug through the intestinal tract has been used to treat chronic kidney disease for several decades. In the past 50 years, clinical researchers have demonstrated that rhubarb-based decoction enema not only ameliorates uremia symptoms, but also reduces blood uremic toxins and beta-microglobulin [7–10]. This treatment has become an indispensable specific therapy for CKD in Chinese medicine (CM); however, studies on its mechanisms have been limited.

On the basis of the theory of the gut–kidney axis [11], animal experiments were designed to evaluate the effect of rhubarb enema on renal tubulointerstitial fibrosis in CKD rats and explore the effect of rhubarb enema on renal oxidative stress and inflammatory injury derived from uremic toxins. The results of the study could provide a new strategy for preventing CKD with colon-targeted Chinese herbs.

## Materials and Methods

### Animals, antibodies, and experimental drugs

Twenty-eight specified pathogen-free, 3-month-old, male Sprague Dawley (SD) rats, were used for this study. Each rat was housed in our animal facility under pathogen-free conditions and fed a standard laboratory diet with free access to water. The temperature was maintained at 18–22°C with a 12-h light/dark cycle. All animal procedures complied with government-published recommendations for the use of laboratory animals. The study was approved by the Institutional Ethics Review Boards of Guangdong Provincial Hospital of Chinese Medicine.

The anti-collagen-I, occludin, 8-hydroxy-2'-deoxyguanosine (8-OHdG), and NF-κB antibodies were procured from Abcam (Cambridge, UK), anti-β-actin antibody was obtained from Sigma-Aldrich (St. Louis, MO, USA). FITC-conjugated anti-mouse IgG, cy3-conjugated anti-rabbit IgG, HRP-conjugated anti-mouse IgG, and rabbit IgG were purchased from Beyotime Biotechnology Company (Shanghai, China).

Rhubarb granules were purchased from Jiang Yin Tianjiang Pharmaceutical Company (Product Lot: 1303196) and were monitored for contaminants (heavy metals, pesticides, and mycotoxins) before formulation.

### Establishing the 5/6 nephrectomized rat model

On the day of the kidney surgery, the 28 SD rats were randomly divided into two groups: sham operation (sham, n = 8) and 5/6Nx (5/6Nx, n = 20). All rats were anesthetized with an intraperitoneal injection of 2.0% pentobarbital sodium (30 mg/kg body weight). For the 20 5/6Nx rats, two-thirds the mass of the left kidney was ablated and a subsequent right unilateral nephrectomy was performed 1.0 week later. For the sham operation group, a laparotomy was performed and the renal pedicle manipulated without any removal of renal mass.

Two months later, the 20 5/6Nx SD rats were randomly divided into two groups: 5/6Nx (5/6Nx, n = 10) and 5/6Nx with rhubarb enema treatment (5/6Nx + rhubarb enema, n = 10). The 5/6Nx + rhubarb enema rats were given an enema with 5.0 mL rhubarb granule solution containing 0.5 g rhubarb granules. The sham operation group and 5/6Nx group rats received the same volume of saline solution by enema. Enema was continued for 1.0 month.

After 1.0 month of rhubarb treatment, the rats were sacrificed and serum was sampled from the abdominal aorta of each. The remnant kidney was decapsulated and divided into several parts. One part was fixed in 10% formalin and processed for histological analysis; another was fixed in optimum cutting temperature compound, quickly frozen in liquid nitrogen, and stored at -80°C for immunofluorescent analysis. The remaining parts were dissected to isolate the kidney tissue, which was quickly frozen in liquid nitrogen and stored at -80°C for protein extraction. Serum creatinine, blood urea nitrogen (BUN) and high-sensitivity C-reactive protein (hs-CRP) were performed in a core clinical pathology laboratory using a standard auto-biochemical analyzer.

### Renal histopathological studies

Sections of the paraffin-embedded tissues were sliced to 2.0 μm thick and stained with hematoxylin and eosin and Masson’s trichrome. The sections were examined in a blind manner. The degree of tubulointerstitial fibrosis was scored as follows: 0, normal tubules with no fibrosis; 1, mild interstitial fibrosis with nearly normal tubules; 2, moderate interstitial fibrosis with some increased thickness of the tubular basement membrane; and 3, marked interstitial fibrosis with diffuse increased thickness of the tubular basement membrane. The tubulointerstitial fibrosis semiquantitative scores of 40 randomly selected fields were summed [12].

### Indoxyl sulfate measurement

The high-performance liquid chromatography (HPLC) tandem mass spectrometry method modified according to Wang and Korfmacher [13] was used to detect IS concentration in the plasma. The detection chemical, including the standard IS potassium salt (purity 99.8%), was obtained from Sigma-Aldrich (St. Louis, MO, USA); the internal standard of hydrochlorothiazide (purity 99.5%) was obtained from Institute for Drug Control (Guangzhou, China).

Briefly, serum samples were deproteinized by adding three parts methanol to one part serum to determine IS. All analyses were performed on an Acquity HPLC system (Agilent Technologies, Santa Clara, CA, USA) and an API3000 triple quadrupole mass spectrometer (Applied Biosystems, CA, USA). A reversed-phase HPLC ZORBAX Eclipse XDB-C18 column (Narrow-Bore 2.1 × 150 mm, 3.5-micron; Agilent Technologies, Santa Clara, CA, USA) was used as a separator. All data were analyzed using Anlyst14.1 (Applied Biosystems, CA, USA). The standard curves were made using the linear regression formula y = 0.0665 x -0.0294 with an R^2^ = 0.9995. The limit of quantification was defined as the lowest concentration for which acceptable precision and accuracy could be guaranteed (<20%). The limit of detection was 0.25 μg/mL.

### Western blot analysis

Kidney tissues were lysed in tadioimmunoprecipitation assay buffer. The lysates were clarified by centrifugation at 12000 rpm for 30 min at 4.0°C, and the protein concentration in each lysate was determined using a BCA Protein Assay Kit (Pierce Protein Biology, Rockford, IL, USA). Protein samples (50 mg) were separated by 6.0–10% sodium dodecyl sulfate polyacrylamide gel electrophoresis and electroblotted onto nitrocellulose membranes. The membranes were blocked with 5.0% skim milk, incubated with a primary antibody at 4.0°C overnight, and then incubated with horseradish peroxidase-conjugated secondary antibody. Immunoreactive bands were visualized using enhanced chemiluminescence reagent and exposure to BIO-RAD ChemiDoc XRS+ system.

### Tissue immunofluorescent staining

Sections were washed twice with PBS for 10 min and preincubated in 10% casein (Vector, Burlingame, CA, USA) in PBS for 30 min. The sections were incubated with primary antibodies overnight in a moisture chamber and then washed sufficiently with phosphate-buffered saline with Tween 20 to remove any unbound antibody. The sections were then incubated with secondary antibody, FITC-conjugated anti-mouse IgG, or cy3-conjugated anti-rabbit IgG for 60 min at room temperature and washed as described for the primary antibody. The sections were mounted on glass slides and analyzed under an Olympus BX61 fluorescence microscope equipped with an Olympus DP72 digital camera.

### Evaluation of 8-hydroxy-2'-deoxyguanosine expression and superoxide dismutase activity

The expression of 8-OHdG in the kidney, a marker of oxidative stress [14], was examined by immunofluorescence using a mouse monoclonal anti-8-OHdG antibody. Superoxide dismutase (SOD) activity was assayed using the Total Superoxide Dismutase Assay Kit (S0101,Beyotime, shanghai) following the manufacturer’s instructions [15].

### Statistical analyses

All data analyses were performed using SPSS 13.0 (SPSS, Inc., Chicago, IL, USA). Data are expressed as the means ± SD. Comparisons among groups were conducted using an analysis of variance. A value of *P* < 0.05 indicates significance.

## Results

### Rhubarb enema improves renal function and inhibits tubulointerstitial injuries

**Fig 1:**
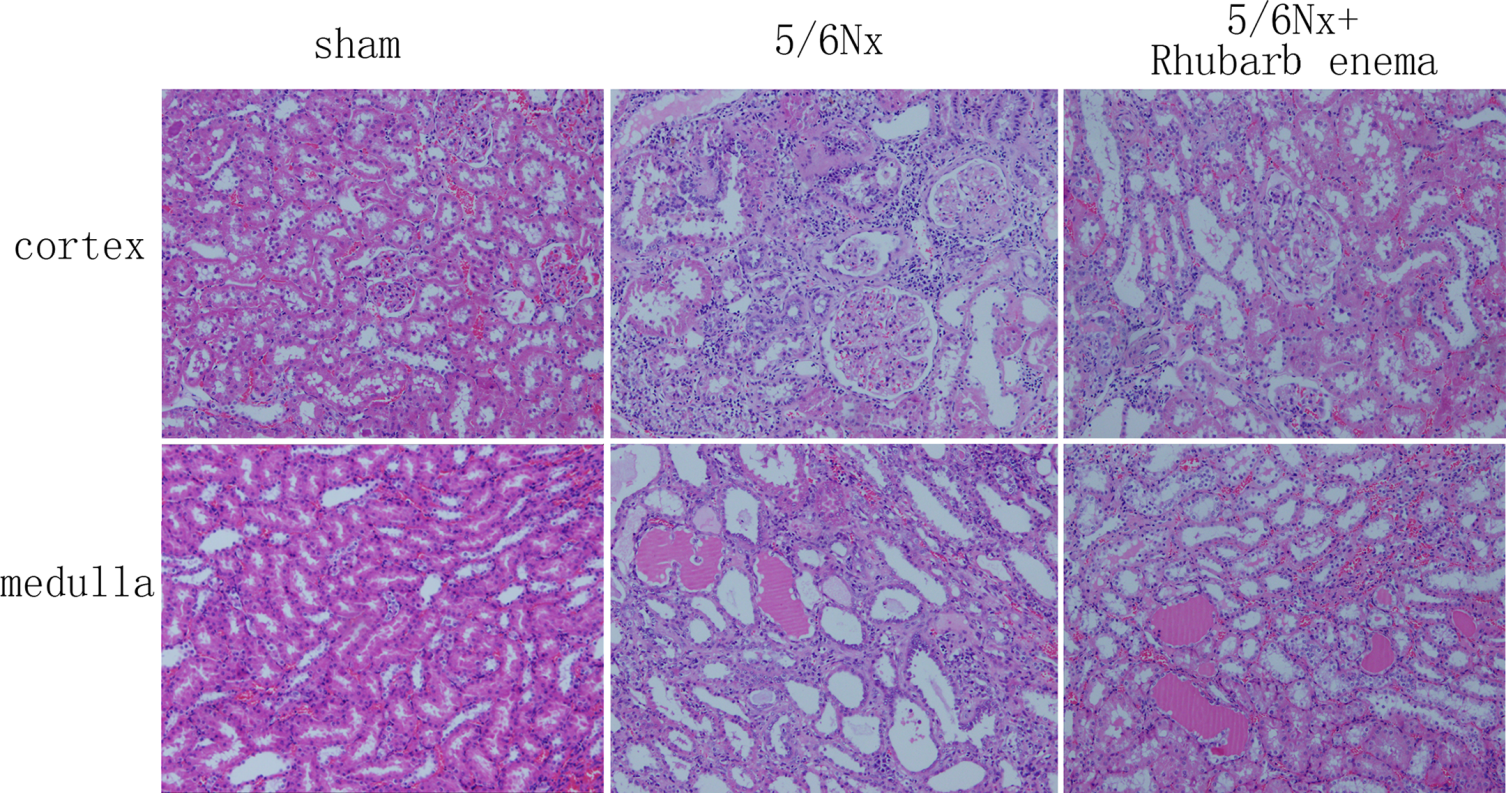
Pathological findings in the sham operation group and the 5/6 nephrectomized rats treated with or without rhubarb enema (hematoxylin and eosin stain, ×100).

Baseline serum creatinine was not different among the three groups. At the end of the study, the sham operation group showed low levels of serum creatinine and normal glomerular and tubulointerstitial morphology. The level of serum creatinine was significantly increased in the 5/6Nx rats; these rats showed enlargement of tubular lumen, protein cast, tubular atrophy, and interstitial expansion accompanied by numerous infiltrations of mononuclear cells and interstitial fibrosis (Table 1, Fig 1).

**Table 1:** Rhubarb enema results among the three groups.

Groups	Scr (baseline, μmol/L)	Scr (the end, μmol/L)
Sham	20.63±1.28	32.83±5.6
5/6Nx	20.54±1.50	71.69±8.75*
5/6Nx+ Rhubarb enema	21.30±1.61	60.09±10.71^#^

*P < 0.05 vs sham operation group.

^#^ *P* < 0.05 vs 5/6Nx group.

The level of serum creatinine in the 5/6Nx + rhubarb enema rats obviously decreased after treatment. The rhubarb enema attenuated the histopathological changes in this group (Table 1 and Fig 1).

### Rhubarb enema attenuates renal tubulointerstitial fibrosis in 5/6Nx rats

**Fig 2:**
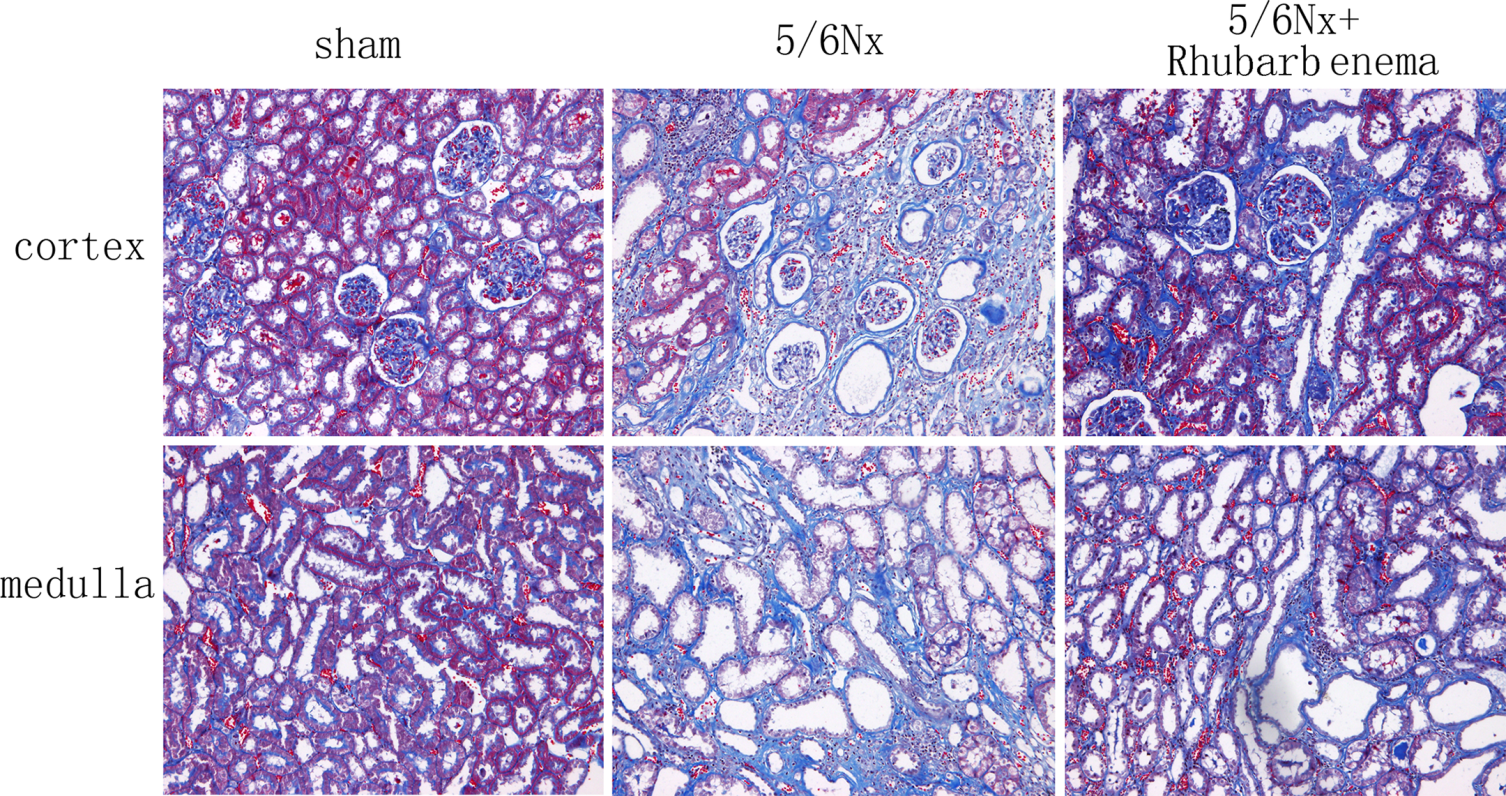
Pathological findings in sham and 5/6Nx rats treated with and without rhubarb enema (Masson’s stain, ×100).

**Fig 3:**
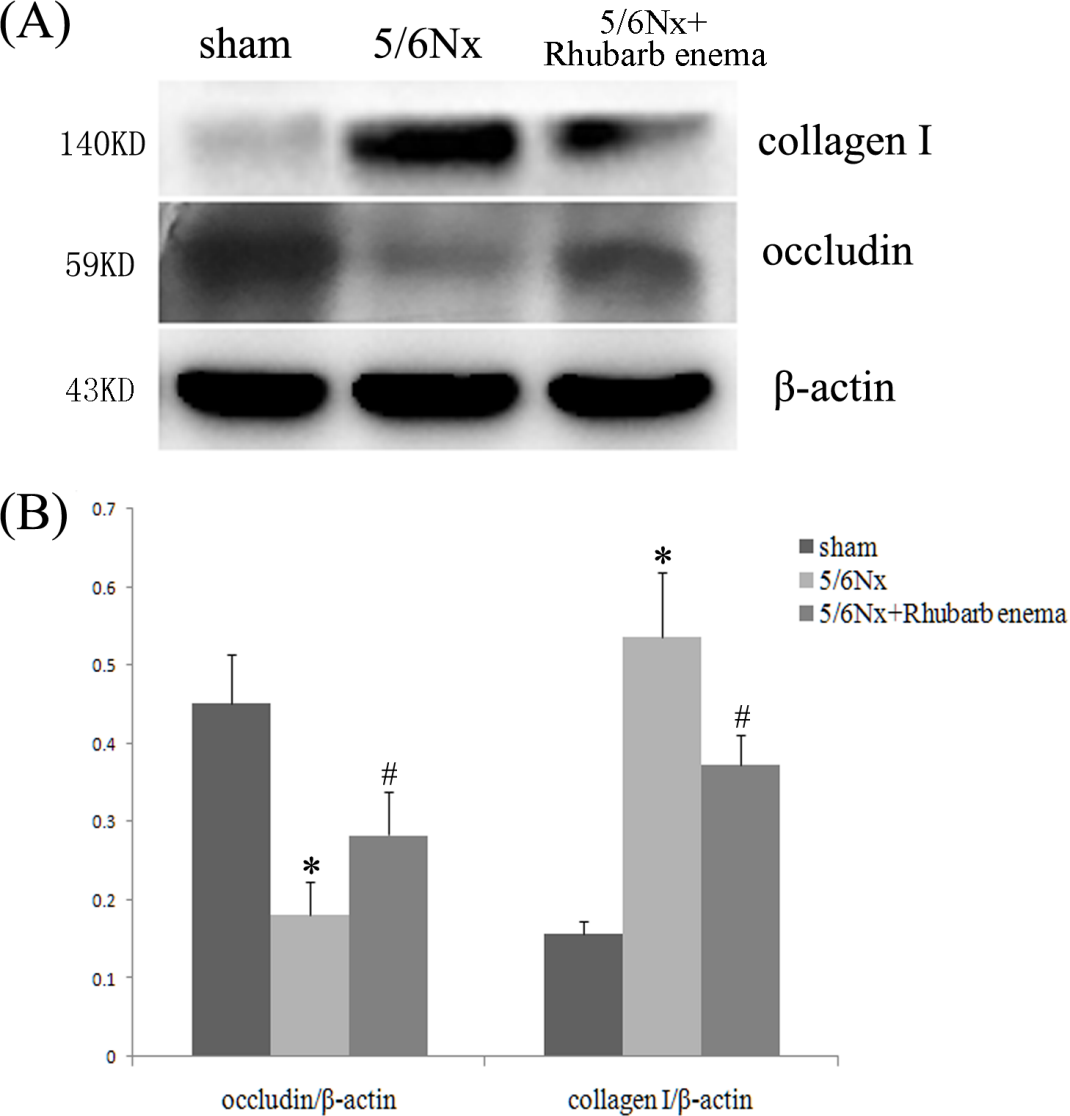
Expressions of occludin and collagen I in kidney tissues. (A) Western-blot analysis of occludin and collagen I in kidney tissues. Lane 1 is the sham operated group, lane 2 is the 5/6 nephrectomized group, and lane 3 is the 5/6 nephrectomized with rhubarb enema treatment group. (B) Occludin and collagen I protein levels. Data are expressed vs. β-actin and were compared by analysis of variance. *P < 0.05 vs. sham operation group. ^#^ *P* < 0.05 vs. 5/6 nephrectomized group.

5/6 Nx rats showed significant tubulointerstial fibrosis as assessed by quantification of the MT-positive area. Four weeks after rhubarb enema treatment, the progression of tubulointerstitial fibrosis was significantly retarded (Fig 2). The tubulointerstitial fibrosis score was reduced by ~18.75% in the 5/6Nx + rhubarb enema rats compared with that in the 5/6Nx rats (*P* < 0.05, respectively, Table 2).The expression of epithelial marker occludin was obviously upregulated and the expression of profibrotic marker collagen I was significantly downregulated in the remnant kidney after the rhubarb enema (Fig 3). In contrast, concomitant treatment with a rhubarb enema significantly attenuated tubulointerstitial fibrosis in 5/6Nx rats.

**Table 2:** Tubulointerstitial fibrosis score.

Groups	tubulointerstitial fibrosis score
Sham	0
5/6Nx	2.67±0.44*
5/6Nx+ Rhubarb enema	2.16±0.55^#^

*P < 0.001 vs sham group.

^#^ *P* < 0.05 vs 5/6Nx group.

### Rhubarb enema reduces serum levels of indoxyl sulfate and hs-CRP in 5/6Nx rats

Substantial evidence supports the accumulation of urine toxins in 5/6 rats. In this study, 5/6Nx group showed significantly increased serum levels of IS compared with that in the sham operation rats, whereas those in the 5/6Nx + rhubarb enema group showed significantly decreased serum levels of IS.

Low levels of hs-CRP were detected in the serum of the sham operation group but was significantly increased in the 5/6Nx group, indicating that urine toxin storage and systemic inflammation appeared in 5/6Nx rats. The serum level of hs-CRP concentration was significantly lower in the 5/6Nx + rhubarb enema treatment group than in the 5/6Nx group (Table 3).

**Table 3:** Serum levels of indoxyl sulfate and high-sensitivity C-reactive protein.

Groups	IS(μg/ml)	hs-CRP(g/g)
Sham	1.13±0.11	0.74±0.18
5/6Nx	2.28±0.52*	15.54±6.35*
5/6Nx+ Rhubarb enema	1.51±0.39^#^	12.17±4.74^#^

IS, indoxyl sulfate; CRP, high-sensitivity C-reactive protein.

*P < 0.05 vs sham group.

^#^ *P* < 0.05 vs 5/6Nx group.

### Rhubarb enema ameliorates renal oxidative stress and inflammatory injury in 5/6Nx rats

**Fig 4:**
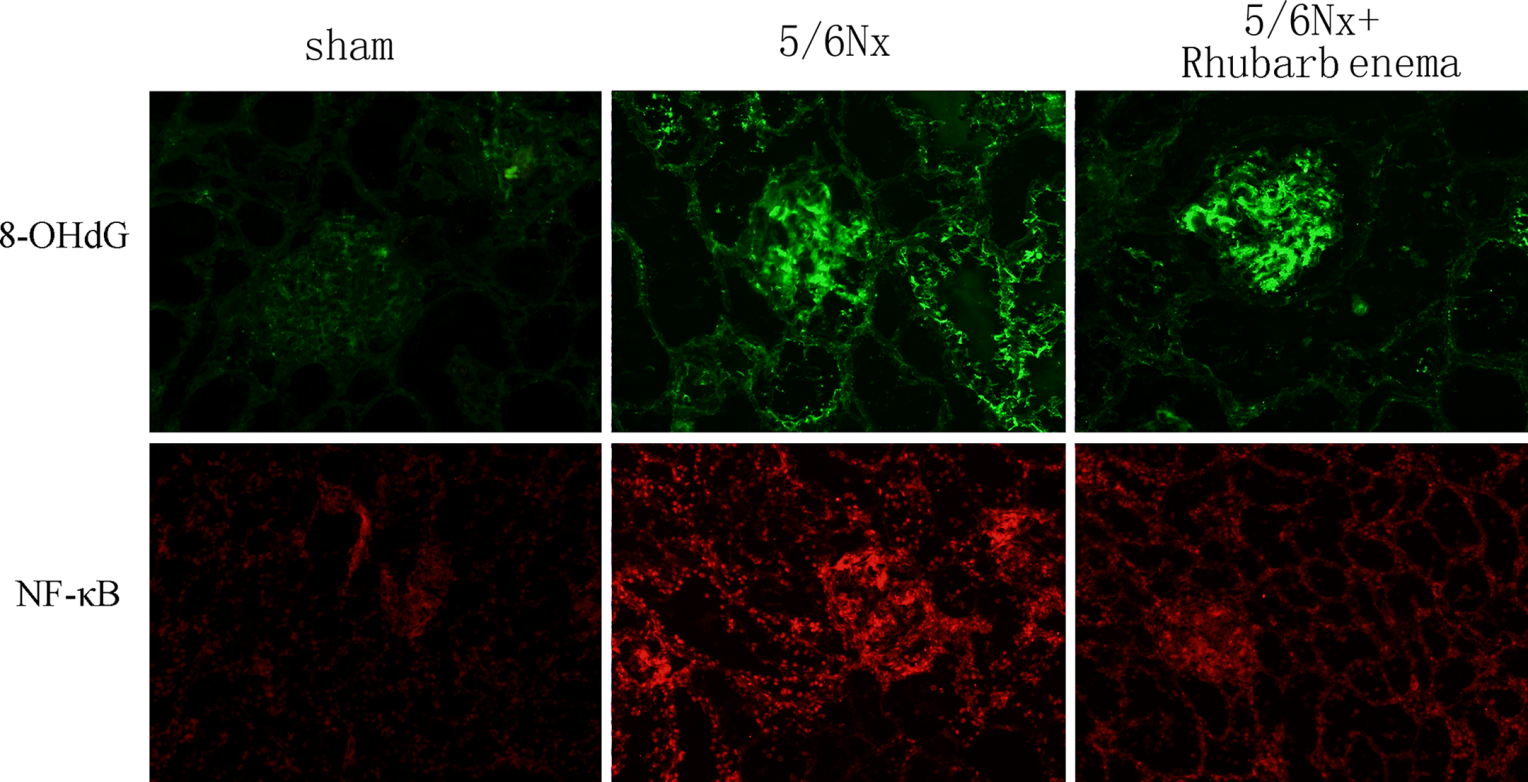
Expressions of 8-hydroxy-2'-deoxyguanosine (8-OHdG) and NF-κB in frozen sections of kidney. Localization of 8-OHdG and NF-κB in frozen sections was determined by immunofluorescence (IF) (×200) with 8-OHdG antibody (green) and NF-κB antibody (red).

**Fig 5:**
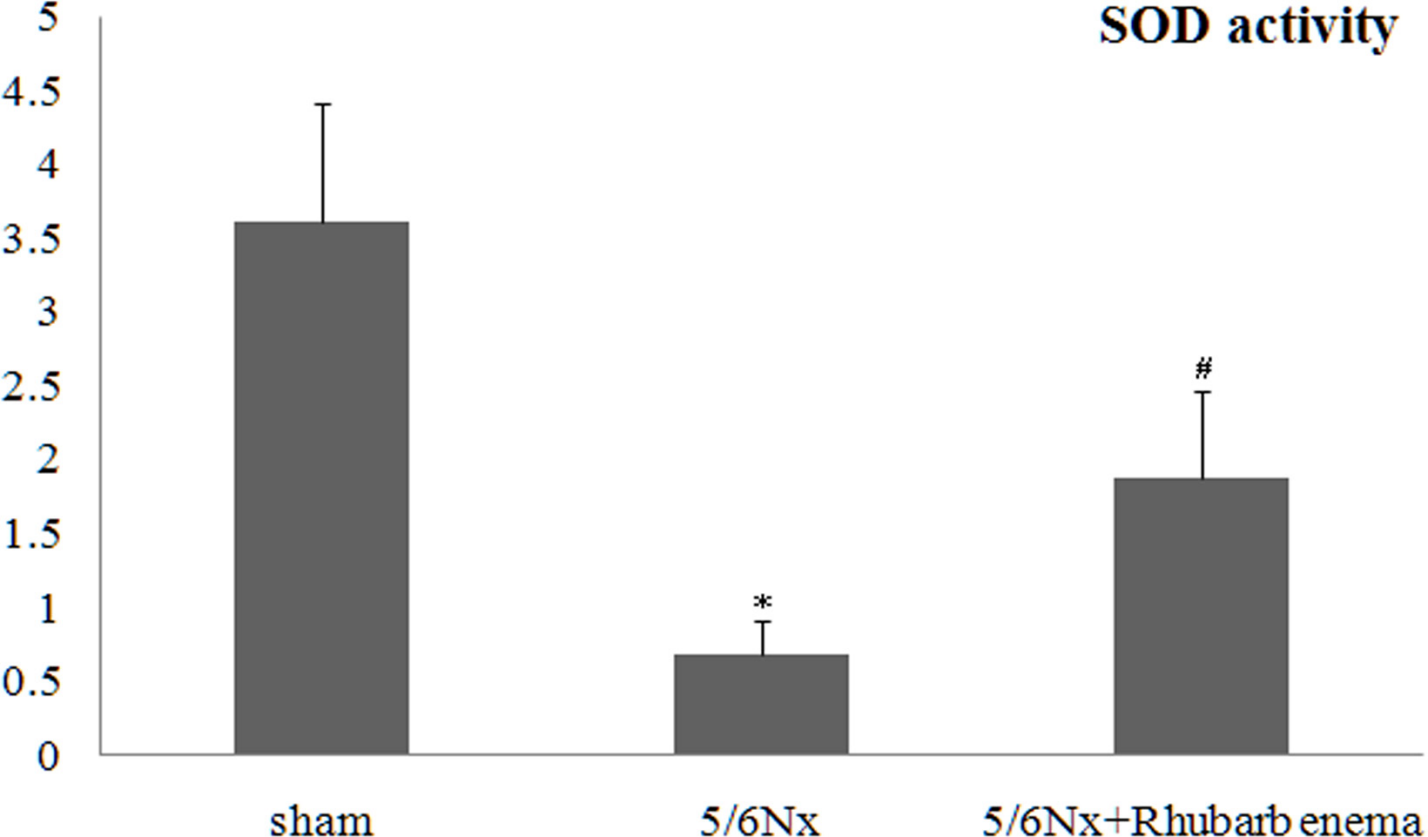
Superoxide dismutase activity assays. Sham operation group rats showed higher SOD values, but SOD activity in 5/6 nephrectomized rats (5/6Nx) was significantly decreased. *P < 0.05 vs sham group. The value of SOD activity in the group treated with rhubarb enema was significantly higher than that in the 5/6Nx group. ^*#*^ *P* < 0.05 vs 5/6Nx group.

**Fig 6:**
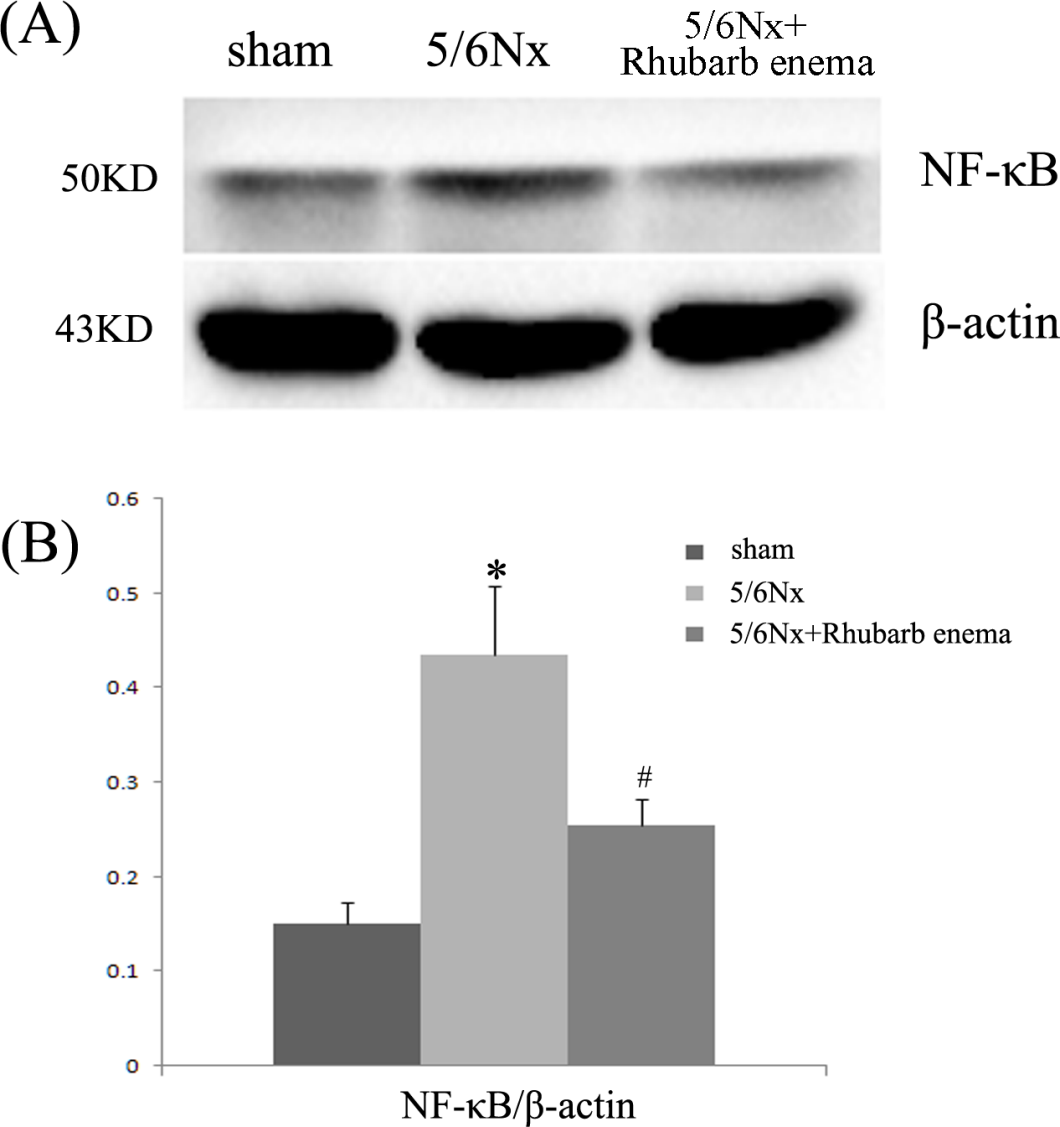
Expression of NF-κB in kidney tissues. (A) Western-blot analysis of NF-κB in kidney tissues. Lane 1 is the sham operation group, lane 2 is the 5/6 nephrectomized group, and lane 3 is the 5/6 nephrectomized with rhubarb enema treatment group. (B) NF-κB protein levels. Data are expressed vs. β-actin and were compared by analysis of variance. *P < 0.05 vs. sham operation group. ^#^ *P* < 0.05 vs. 5/6 nephrectomized group.

Renal tissue oxidative stress was evaluated using 8-OHdG immunofluorescent staining, a critical biomarker of oxidative stress and SOD activity assays. Renal inflammatory injury was evaluated according to the level of NF-κB. 5/6Nx rats showed markedly increased 8-OHdG-positive areas in both the glomeruli and tubules (Fig 4). Increased 8-OHdG staining was associated with decreased SOD activity (Fig 5). Expression of NF-κB was detected by immunofluorescence staining and western blot. The expression of NF-κB was significantly increased in both the glomeruli and tubules (Figs 4 and 6). Rhubarb enema treatment cannot attenuate the 8-OHdG expression in the glomeruli, but can attenuate its expression in tubules and can enhance SOD activity in the remnant kidney. Concurrently, rhubarb enema treatment also decreased the expression of NF-κB in the remnant kidney, especially in the tubules (Figs 4–6).

## Discussion

The present study demonstrated that rhubarb enema inhibits renal tubulointerstitial fibrosis in 5/6Nx rats, accompanied by reduced serum levels of IS, and ameliorates renal oxidative stress and inflammatory injury. The evidence supports that the effect of rhubarb enema might be because of its suppression of serum levels of IS.

Evidence suggests that the accumulation of a uremic toxin, especially IS, is a key mechanism in cardiovascular disease in CKD [16, 17]. IS is related to cardiovascular mortality [18, 19] and can damage the kidneys and cardiovascular system, which can lead to the progression of CKD and an increase in cardiovascular events and can result in higher mortality of patients with CKD [20, 21]. This study also demonstrated that IS promotes the continued deterioration of kidney function by inducing oxidative stress, producing a variety of inflammatory cytokines, and promoting interstitial fibrosis in kidney tubules [22].

Recent studies found that the colon is an important organ from where uremic toxins are generated. In CKD patients, an imbalance occurs mainly in the colon, presenting as a decrease of probiotics, such as *Lactobacillus* and *Bifidobacterium* and an increase in pathogenic bacteria, such as *Escherichia coli* and *Enterococcus* [23, 24]. Under these conditions, bacteria (mainly, *E*. *coli*) produce dozens of toxins from glycolysis of retained proteins. For example, part of tryptophan is metabolized into indole by *E*. *coli* in the colon, which is absorbed into the blood through the intestinal wall and metabolized into IS in the liver [11]. IS is a protein-bound toxin with a molecular weight >500 D and difficult to remove [25]. How to reduce the production of gut-derived toxins such as IS is a critical issue.

CM practitioners, including famous CM masters in nephrology, have come to a consensus that some special uremic syndrome related to the toxins from colon is alleviated by rhubarb enema treatment [26]. Modern pharmacological researchers have also demonstrated that rhubarb promotes the excretion of toxic metabolites, such as BUN and phosphorus [27]. Some experts claim that rhubarb in the colon can regulate intestinal flora and reduce intestine-derived uremic toxins produced by gut bacteria, which provides new targets for delaying CKD progression [28]. Other studies showed that rhubarb can also directly promote intestinal dynamics [29], protect intestinal barrier function, and regulate intestinal flora [30].

We thus have hypothesized that rhubarb enema can accelerate intestinal dynamics, improve intestinal function, and reduce the production and absorption of intestine-derived uremic toxins, such as IS, and promote their excretion, which might reduce renal fibrosis and delay renal progression.

Our study demonstrates that rhubarb enema inhibits renal tubulointerstitial fibrosis in 5/6Nx rats, accompanied by reduced serum levels of IS, and can ameliorate renal oxidative stress and inflammatory injury. Our data have shed new light on the mechanism by which rhubarb enema treatment elicits its renoprotective action in the pathogenesis of tubulointerstitial fibrosis.

## Supporting Information

S1 Appendix Animal ethics of the study. (PDF)

S2 Appendix Animal ethics of the study (in English). (PDF)

S1 Data Baseline serum creatinine of animals. (XLSX)

S2 Data Experimental data of the study. (XLS)
