# Supplementary material for: Rhubarb Enema Attenuates Renal Tubulointerstitial Fibrosis in 5/6 Nephrectomized Rats by Alleviating Indoxyl Sulfate Overload
Source: PLoS One. 2015 Dec 15;10(12):e0144726. doi: 10.1371/journal.pone.0144726 (PMC4684395; doi:10.1371/journal.pone.0144726)
Supplement: S1 Appendix — (PDF) [file pone.0144726.s001.pdf]

广东省中医院实验动物伦理委员会对动物实验研究的审查结果

一、实验项目基本情况：

实验项目名称	基于肠肾轴理论模型探讨大黄结肠给药改善肾间质纤维化的机制研究	编号	2013011
动物来源		品种品系	SD 大鼠
申请单位			
联系人	邹川	联系电话	
实验目的	基于肠肾轴理论模型探讨大黄结肠给药改善肾间质纤维化的机制研究		

二、伦理委员会讨论内容：

参加动物实验研究者资格：	1、符合条件	2、不符合条件	
提供的动物伦理审查材料：	1、符合条件	2、不符合条件	
动物实验的必要性：	1、必要	2、不必要	
是否符合动物福利原则：	1、是	2、否	
动物实验的环境条件是否符合国家标准：	1、是	2、否	
实验方案：	1、合理	2、基本合理	3、不合理

三、伦理委员会审议情况：

应到会委员数：9人	实际到会委员数：5 人	未到委员及原因：
会议地点：		

四、审议结论：

同意实验

广东省中医院 广东省中医药科学院 实验动物伦理委员会

曾 星	
丘小惠	
邓时贵	
李慧	
孙景波	
佘谊君	
何建强	
韩凌	
郑广娟	

主任或副主任签名	

2013 年 2 月 11 日
