# Supplementary material for: Rhubarb Enema Attenuates Renal Tubulointerstitial Fibrosis in 5/6 Nephrectomized Rats by Alleviating Indoxyl Sulfate Overload
Source: PLoS One. 2015 Dec 15;10(12):e0144726. doi: 10.1371/journal.pone.0144726 (PMC4684395; doi:10.1371/journal.pone.0144726)
Supplement: S2 Appendix — (PDF) [file pone.0144726.s002.pdf]

Review result of Animal Study performed by Animal Ethical Review Committee of Guangdong Province Hospital Traditional Chinese Medicine

1. Basic Information of the Research Project

Project Name	The study on the mechanism of improving renal interstitial fibrosis with rhubarb enema based on the theory of the gut-kidney axis	Number	2013011
Source of animals		Species or strain	SD Rat
Insititue of Application			
Contact Person	Chuan Zou	Phone number	
Purpose of Study	Exploring the mechanism of improving renal interstitial fibrosis with rhubarb enema based on the theory of the gut-kidney axis		

2. The Content of the Ethical Review

Qualifications of the Researchers of Animal Study	1✓ Accord with regulations	2、Not accord with regulations	
Documents for ethical review	1✓ Accord with regulations	2、Not accord with regulations	
Necessity of animal study	1、Necessary ✓	2、Not Necessary	
Accordance with the principle of animal welfare	1、Yes	2、No	
Accordance of the animal environment with the national stardard	1✓ Yes	2、No	
Research plan	1✓ Very Reasonable	2、Basically reasonable	3、Not Reasonable

3. The Record of the Ethical Review Meeting

Number of committee members supposed to come: 9	Number of committee members actually come: 5	Reason for absence
Meeting Location		

4. Conclusion of Ethical Review

同意实验

Approve this study

Seal of the Animal Ethical Review Committee of Guangdong Province Hospital Traditional Chinese Medicine

曾星	[signature]
丘小惠	
邓时贵	[signature]
李慧	[signature]
孙景波	[signature]
佘谊君	
何建强	[signature]
韩凌	
郑广娟	

Signature of Chairman or vice chairmam	[signature]

2013 year 2 month 11 day
